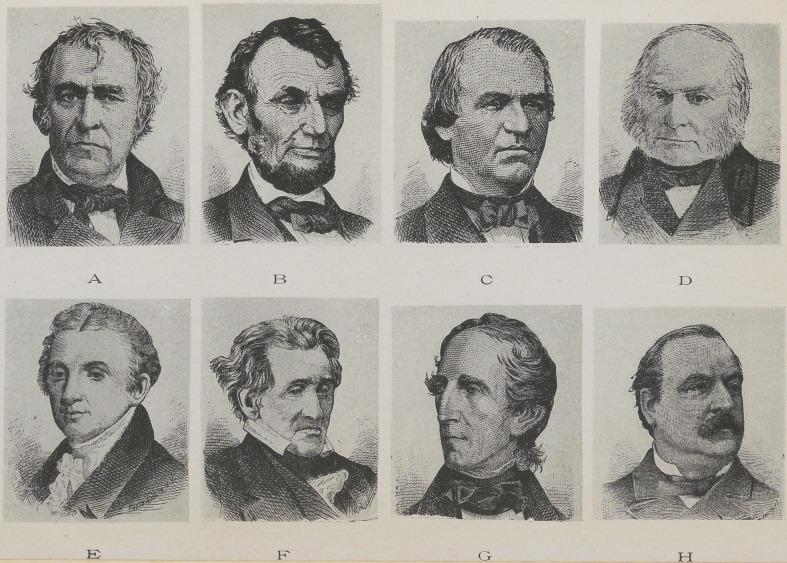# The Basal Temperament Relative to Prosthesis

**Published:** 1901-12-15

**Authors:** B. J. Cigrand


THE
### DENTAL REGISTER.

Vol. LV. December 15, 1901. No. 12.

### THE BASAL TEMPERAMENT RELATIVE TO PROSTHESIS.

BY B. J. CIGRAND, B.S., M.S. D.D.S.

Read at the Bi-State Dental Convention of the Northern Indiana and Southwestern Michigan Societies, September 25th, 1901.

For years prior to my affiliation with the dental fraternity I had been a student of physiognomy and kindred sciences which reveal the varied characteristics of man. Aside from dentistry, few subjects afford me more pleasure than the history of the advancement and character of the human family, and I must confess that the profession in which we are engaged well affords an opportunity for inquiry into the general dispositions of the people ; for it is in the practice of our profession that we come in direct contact with all classes, conditions and races of humanity.

I am impelled to say in the beginning of my paper that it matters little how learned or proficient one may be in the general practice of the principles of dental science, if he be ignorant or regardless of the general character or idiosyncracies of the patients for whom he operates he most certainly will be a colossal failure as a dentist.

In an article by Dr. J. N. Crouse this very important remark occurs: "Human nature—that subtle, indefinable, hard-to-understand something which reveals itself constantly in various shapes—this human nature must be met, understood and controlled before a man can become a successful dentist in the full sense of the word. Every dentist with any amount of practice finds all sorts of dispositions and temperaments to deal with —the pleasant and the disagreeable, the good-natured and the cross, the reasonable and the unreasonable, the honest and the dishonest, the generous and the selfish, the appreciative and the unappreciative; the nervous people out of health, the hysterical subjects, the old maids and bachelors who are seldom well pleased, and the capricious, self-willed and ungoverned children—all of these must be met and dealt with under the most trying circumstances." He then says: "A distinguished educator has said that 'all education can be summed up as knowing yourself, knowing your fellow-man, and knowing how to adapt yourself to your fellow man.' A dentist should be a good judge of human nature and be able to control his various patients with their different peculiarities, and this requires more than an average intelligence. Much, however, can be acquired by practice and study of the subject. This important faculty is so closely related to the various qualifications of a dentist that its advantage can readily be seen."

Other distinguished men of the dental profession have emphasized similar thoughts. Prominently among them are Drs. Kingsley, Allport, Harris and Richardson. I might mention scores of others eqally eminent, but I well know that there is not present here a practitioner who does not fully realize the importance of a practical knowledge of temperaments and facial outlines.

In dental prosthesis and oral surgery the modern dentist must be, in the full sense of the word, a "facial sculptor," for to his tender care and consideration is left the moulding of many a scowl or smile. He must appreciate the lines of beauty in expression and discern at a glance the changes necessary in the different physiognomy to make them charming and inviting rather than repellant and false. It is in the practice of prosthesis that the study of the face is most essential, especially so since "restoration" is its distinctive feature. But he needs a fair understanding of the principles of human character, aside from the purpose of restoring, for if he wishes to attain an agreeable result in any operation he must also know whom he is working for.

The face is divine territory and should receive the closest consideration of all conscientious practitioners. The face is in truth the window of the brain, the avenue to the mind and the symbol of the character. The face is the servant of the emotions; it mirrors the feelings and gives expression to the impulses. The face is the visible record, the map of the heart proclaiming the character of the individual to all who can read. Upon it are reflected the atoms of individuality which make up the career of its owner. It is the herald which advertises the true composition of the man and tells in unmistaking language the traits, peculiarities and characteristics of the person wearing it. These symbols which we so closely trace on the face are not occult or secret, but are open and plain, so much so that even a child may know distinctly the heart of its possessor.

The changing expression of the face is universally regarded as giving response to the passing thought or emotion of the mind. Thus, if one be long afflicted by grief or blessed by joy, wearied by trouble or vexed with care, shadowed by melancholy or excited by wit, inspired by faith or led by delusions, inflated by pride or subjected to shame—the emotions awakened by these different states reveal themselves in the face and become so fixed as to defy concealment. But let one's circumstances become suddenly reversed ; let grief be converted into joy ; let oppression be turned to liberty, and the lines of care and vexation will fade away and scarcely a trace will remain to tell the tale. In short, the sunshine and shadow of our lives are correspondingly engraven on the moldable face. And it is this ever and constant change of the face that demonstrates the two-sidedness of man's being. It proves beyond the shadow of a doubt that all men are subject to development or the reverse ; it also clearly shows that all men —and I mean women, too—have a two-fold or dual character, the one being natural and the other acquired. The dual character is emphatically rspresented in Robert Louis Stevenson's novel entitled "Dr. Jeckyl and Mr. Hyde." Be the acquired character what it will, the innate or created one is ever the power behind the throne. The Germans have a saying which is true to the test, namely, "What the Gods have given you the winds will not blow away."

But it is the innate or natural dispositions of persons that we as dentists must most clearly acquaint ourselves with, since while engaged in our work we operate on nature's being, and any pain or discomfort which we inflict has an immediate response, a response so sudden that it is almost beyond the power of the patient to control. I do not wish to be understood as meaning that a keen knowledge of the acquired disposition is needless, for we must be well versed in both the innate as well as the acquired characters if we desire to accomplish successfully our task and at the same time keep in good humor the acquired character of the patient. A safe rule to follow is this: "Keep on the dexter side of the innate character."

Now, having given a few general obserxations on the certainty and usefulness of an understanding of natures, I will endeavor to briefly tell what indices of character are revealed on the lower third of the face; and let me assure you that the deductions which I here present have been evolved from personal observation, guided by the safe rule of comparison. Much of what I know on the subject *must of necessity* come from reading and research, but the conclusions which I have drawn are uninfluenced by pre-existing theories; nor have I projected a theory and then gone forth to find material to prove it true, but I have made it a point to collect all possible notes, observe closely and compare freely, before permitting myself to formulate a theory or conclusion.

Anatomists and physiologists differ widely as to the definition of the face. There are those who include the forehead as a part of the face, and there are others who claim that the face begins at the supercilliary ridges and embraces the eyes, nose, cheeks, mouth and chin. I prefer the former definition, which holds that the forehead is inclusive in the face. Dividing the face into three equal parts, we have in the lower third the lips, mouth, chin, and greater portion of the cheeks and inferior maxilla.

*Lips and Mouth.*—Dr. Oliver W. Holmes says:

"All parts of the face doubtless have their fixed relations to each other and to the character of the person to whom the face belongs, but there is one feature which more than any other facial sign reveals the nature of the individual. This feature is the mouth, and the portion of it referred to is the corner. A circle of half an inch radius, having its center at the junction of the two lips, will include the chief focus of expression."

The tongue may be silent, but the mouth and lips never cease to speak; they are ever translating to intelligent observers the inner life of the owner.

A long under lip, which when viewed laterally, as in Figs. 1 and A, indicates firmness, when perfectly straight shows a tendency toward stubbornness. The saying, "he has a stiff upper lip," is very appropriate when it is intended to convey that the person is firm and unyielding. People possessing this kind of a lip never surrender, and meet the assaults of adversity or the attacks of enemies as the rock meets the surging waves. A large number of generals have lips of this character, Gen. Zachary Taylor being prominent among them. When the lip curves in at the junction of the lips, as in Fig. 2, it is a mark of determination and is usually seen in explorers and investigators. A short upper lip, as in Fig. 3, portrays a weak and wavering character. People with this symbol are constantly changing their minds and have hardly enough stability and fixedness of purpose to gain a livelihood. They are the "I can't" element in the race, or, as the French say, "He's like the weather."

When the upper lip is curved gracefully outwards, as in Figs. 4 and B, it symbolizes ambition. These persons evince a good degree of self-respect and dignity. You find them preferring the place of a leader rather

Figure I
Fig II
Fig III
Fig IV
Fig VI
Fig VII
Fig VIII
Fig IX
Fig X
Fig XI
Fig XII
Fig XIV
Fig XV

than that of a follower. They are seldom overbearing and are usually of a sunny disposition ; they delight to be in society and enjoy a happy "social chat" ; they have an agreeable and fascinating manner and an acceptable way of saying and doing things. Abraham Lincoln possessed this lip and was true to its significance.

An upper lip which is short and fails to cover the teeth and gums, as in Fig. 5, indicates approbativeness. This lip is seen in people of a vain disposition ; they are mortified by censure and greatly elated by words of commendation ; they are too fond of praise and delight in being flattered ; to blame them wounds their feelings beyond reasonableness. People of this index are so sensitive to criticism that they shun public office or any great public trust. Imagine a Cæsar or Napoleon with a short upper lip, half open mouth. Could you dream of Jefferson, Franklin or Lincoln with this gaping mouth? These lips no man had who served in the armies of Napoleon, for he made it a rule to promote no man who could not close his mouth.

The lips of contempt or scorn are familiar to us all, but to be certain that you may recognize this pessimistic tendency I designed Figs. 6 and C. Contempt protrudes the lower lip and draws down the corners of the mouth. People of this lip are naturally inclined to fault-finding ; they long to provoke the ill-will of those about them ; they delight to abridge the pleasure of their friends, and have a cold, repulsive and antagonistic being. Andrew Johnson possessed many of these traits.

The thin and illy defined lip, as seen in Figs. 7 and D, denotes a cold, unloving nature ; industry, love of order and precision are there in unmistaking language.

This class of people are unsocial and prefer to live to themselves; it bores them to have companionship with any one; they are given to seclusion; they form but few attachments and manifest but a sparing degree of affection for any person. They are the class of people who have no time for recreation and are incessantly employed. A lip of this character we see in the face of John Quincy Adams.

When the lips are full and well rounded, as in Figs. 8 and E, it exhibits the fact that affectionate and most loving thoughts engage the mind. Mothers who love their children to such a degree as to approximate worship have invariably this lip. This lip is most common to the female sex. Warm-hearted sympathy is its potent language. Though these lips signify that their possessor worships Cupid, they nevertheless are emblems of purity and are seldom seen on vicious and crouching countenances. The model mouth of the Greek Venus is of this beautiful shape. James Monroe displays these lips, and his tender love for those about him corroborates the silent voice of his mouth.

The mirthful lip is well proportioned; the middle line is equally serpentine on both sides of the median line, as in Fig. 9. This lip seems to be as the Germans say, "limber and quick," and is usually accompanied by a glib tongue. Voltaire's lip is a familiar illustration.

*Chin and Jaw.*—You have all recognized the great variety which exists in the form and quality of the chin. It may be prominent or retreating, long or short, round or pointed, square or dimpled. Few attach any significance to the shape of the chin, supposing that its shape is merely accidental, but those of you who have given the matter serious thought will assert that the chin demonstrates characteristic peculiarities. One of the marked differences between man and beast is the fact that the man has a chin, while the animals present no such development. We have all noticed that a chinless person is weak-minded and bordering on idiocy or imbecility; this is certainly strange, yet nature's symbols must bow down to nature's command.

The prominent chin, as in Figs. 10 and F, always depicts a positive character and can be seen on persons noted for resolution, perseverance and executiveness. These people are prone to control and command others and to make external circumstances bend to human powers. Courage and fortitude are here clearly stamped. They are people who usually substantiate by argument or force what they declare. They are the persons who enter a battle to "fight to a finish," and the fight is never finished until they are the victors. This chin is noticeable on Andrew Jackson, who was well known as a fearless soldier and statesman.

The sharp and prominent chin, as in Fig. 11, is generally indicative of an inquisitive and crafty nature. This class of people are apt to take an undue interest in other persons' affairs; you will generally discover them in gossip circles and invariably find them to be newsmongers. They are somewhat quick-tempered and manifest a super-sensitive nature. They are painstaking investigators, delighted to pry into social matters; they are natural born detectives and gratify themselves most decidedly in shadowing the suspicious people of the vicinity. The eminent Frenchman, Cardinal Richelieu, approaches this character.

The receding chin, as in Fig. 12, betokens that the owner lacks executive force; want of will power is his failing. These people have no mind of their own and are completely under the subjection of some trusted friend. When this chin is accompanied by a short upper lip, as in Figs. 3 and G, and the forehead is receding, it is a safe criterion of weak-mindedness. George I. of England may safely be classed as typifying this character. His retreating chin and forehead and the partially open mouth are familiar to all students of history. President Tyler gave evidence of a similar nature. The chin and lower jaw are inseparably associated in the study of the lower third of the face. They contribute very liberally toward giving an expression to the upper two-thirds of the countenance and are the seat of many characteristic symbols. There are three varieties of shapes common to the inferior maxilla which are deserving of our attention. These are the angular jaw, the round or bulldog jaw, and the infantile jaw. The angular jaw, as illustrated in Fig. 13, is so called on account of the acute angle which is formed by the union of the ramus and the body of this maxilla, and it is surprising how few matured individuals present this well-proportioned jaw. This jaw is most common in man, and it is an earnest indication of firmness and determination. Men with this jaw often pursue their ends with a reckless yet stern disregard for their physical welfare; nothing can turn them aside from their purpose, and they attain their success by means of their great energy, perseverance and endurance, rather than by forethought or deep scheming. They are men of the field rather than of the chamber. They are observers rather than thinkers, and know no word like fear. Their will power is most active and they are the acknowledged leaders in the sphere of active life. As speakers they use strong expressions, emphasize many words and talk to the point. They have no time for sentiment, scarcely appreciate fine art, and laugh at the

A

B

C

D

E

F

G

H

jingle of poetry. To them this is a matter-of-fact world with no time for rest. Their motto seems to be "to do or die." The great Roman, Julius Cæsar, had such a jaw, and his life of conquests bears witness that he was true to his nature. The round or bulldog jaw, as shown in Figs. 14 and H, is usually seen on persons of pugilistic tendencies, though it is quite common to the stern business man. It portrays a tenacious, selfish and decisive character. These people are of an antagonistic disposition, finding great pleasure in wielding their power of "fists" against some opposing human force. The prize-fighter's countenance is too clearly seen by your mind's eye to need further description. Ex-President Cleveland bears a most striking resemblance to this face.

The infantile jaw, as Dr. Oliver Wendell Holmes named it, is seen in Fig. 15. He designated it thus from its analogy to the undeveloped jaw of the infant, having an obtuse angle. People with this jaw exhibit sly and fox-like propensities. They have great will power, but very little courage. They prefer to command circumstances rather than men ; they are reserved in the expression of their feelings ; they keep their affairs, plans or designs to themselves ; are discreet and delight in concealment. They have mysterious, tricky, deceptive and shrewd methods in performing any task. If, when engaged as generals, they are inclined to practice strategy, they much prefer indirect approaching to a straightforward or open field encounter.

No matter how anatomically correct or how skillfully adapted for speech and mastication an artificial denture may be, yet if it bear not the relation demanded by age, facial contour and temperament, it can not be otherwise than that its artificiality will be apparent to every beholder. The law of harmony thus found in nature, between the teeth and other physical characteristics, require due respect to size, shape, color and other qualities in an artificial denture in order that it shall correspond with other indications of temperament. There is no dental service that, from the æsthetic standpoint, is as a rule so illy performed as the prosthetic.

*The Motive Temperament.*—To the ancients it was known as the bilious temperament. Persons of this quality of body and mind are "human powers," strength and duration their peculiar characteristics, constitutional powers and great muscular strength. Tall and angular, active, steady and firm. Facial contour, square, angular and high cheek bones. Complexion, dark and sallow. Quality of voice, strong and full of vibration. Nose, strong and usually Roman. Eyes, dark and piercing. Hair, dark, coarse and abundant. Fingers, long and knotty. Favorable characteristics, energetic, persevering, executive and ambitious. Unfavorable characteristics, extreme in expression and often domineering. Examples of type, Old Roman. Comparitive anatomy, the lion. Human example, Daniel Webster.

*The Mental Temperament.*—To the ancients known as the sensitive temperament. This temperament might be called the nervous, refined or brainy. External indications, well developed nervous system, studious and refined expression. Constitutional outline, full and graceful figure. General movements, quick, active, decided and restless. Facial contour, delicate, oval and finely cut. Complexion, abounding in grayish tint, lack of flush. Quality of voice, strong, clear, high pitched and melodious. Nose, well developed, usually Grecian. Eyes, bright, expressive, usually gray or blue. Hair, not abundant, and fine in texture, usually brown. Fingers, long and thin. Favorable characteristics, refined, imaginative, scholarly and studious. Unfavorable characteristics, sensitive, aspiring, and often eccentric. Example of type, American people. Comparative anatomy, the greyhound. Human example, Thomas Jefferson.

*The Sanguine Temperament.*—To the ancients known as the plethoric temperament. This temperament might be called the sanguine, hyperæmic or cardiac. External indications, flush and florid complexion. Constitutional outline, medium in height and lively, general movements active and easy. Facial contour, round, with full forehead. Complexion, florid and animated. Quality of voice, soft and clear. Nose, rather small, usually Grecian. Eyes, usually blue. Facial contour, round and forehead unshapely, complexion pallid and muggy. Quality of voice, poor and indistinct. Nose, small. Eyes, sleepy, inexpressive, generally sluggish. Hair, blond and sparing. Fingers, medium in length and rounded. Favorable characteristics, mirthful, social and friendly. Unfavorable characteristics, passionate and high-tempered. Examples of type, Danes and Germans. Comparative anatomy, shepherd dog. Human example, James A. Garfield.

*The Lymphatic Temperament.*—To the ancients known as the phlegmatic. This temperament might be called stomachic or digestive. External indications, round and well-developed jaws. Constitutional outlines, fleshy and bulky, general movements slow and sluggish. Hair, coarse, straight and drab. Fingers, short, flabby and cold. Favorable characteristics, contented, agreeable and jolly. Unfavorable characteristics, sluggish, lazy and unenergetic. Example of type, Esquamaux.

Comparative anatomy, swine. Human example, Henry VIII. of England.

In concluding this paper, privilege me to remind you of the importance of a fair understanding of the various characters which reveal themselves on the human face, and permit me to impress you with the fact that the investigating and searching elements of the coming generations will devote a liberal portion of their time in the attempt to solve the inviting mysteries of psychology, physiognomy and other co-related subjects; the mental as well as the physical characteristics of man will engage the undivided attention of the rising humanity, and the wonderful deductions which are yet to come to light relative to mental phenomena will be as profound as any discoveries ever made.

Matters foreign to the peace and general welfare of our race can claim but little time of the coming scientists, for man is awakened to the truth of Pope's remark, "The proper study of mankind is man." And the dentists and physicians can not remain dormant or regardless of this great truth if they hope in fact to be the benefactors to suffering humanity.

## Figures and Tables

**Figure f1:**
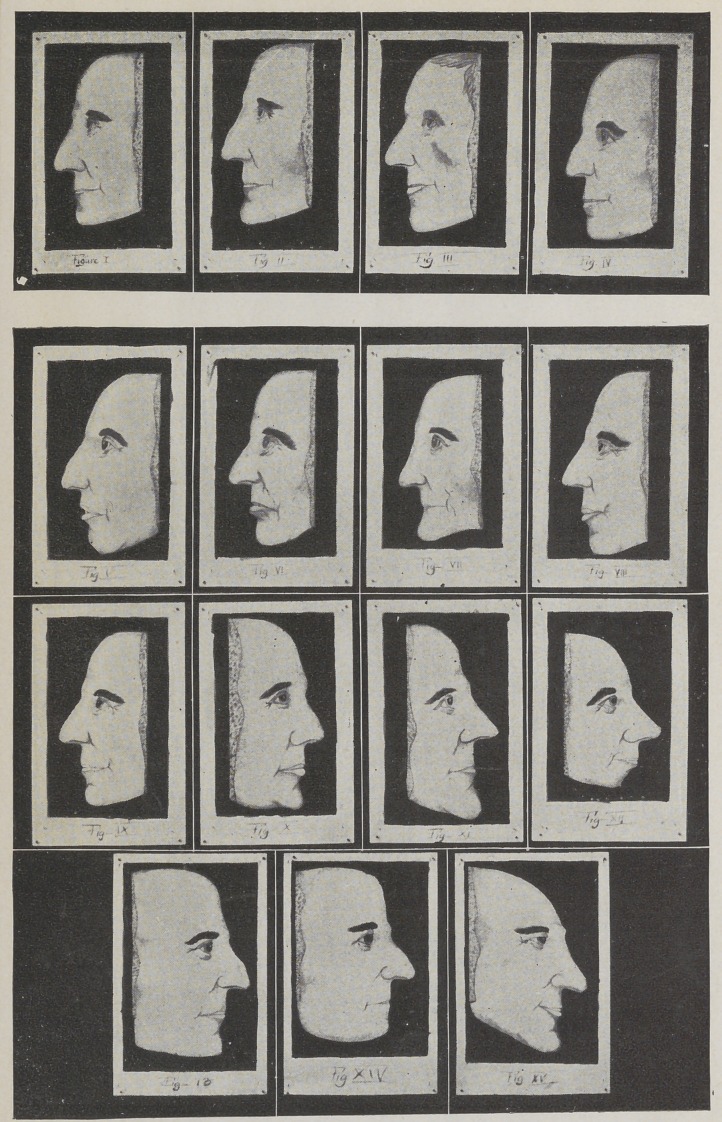


**Figure f2:**